# Phospholipid‐Based Carriers Improve the Stability of *Cyclopia subternata* Phenolic Compounds in a Beverage Matrix, While Modulating Their Passive Diffusion Across a Simulated Intestinal Membrane

**DOI:** 10.1111/1750-3841.71630

**Published:** 2026-11-11

**Authors:** Jade Tobin, Chantelle Human, Dalene de Beer, Marieta van der Rijst

**Affiliations:** ^1^ Plant Bioactives Group, Post‐Harvest and Agro‐Processing Technologies Agricultural Research Council Infruitec‐Nietvoorbij Stellenbosch South Africa; ^2^ Department of Food Science Stellenbosch University Stellenbosch South Africa; ^3^ Biometry Unit Agricultural Research Council Stellenbosch South Africa

## Abstract

Phenolic compounds are susceptible to degradation during food storage and gastrointestinal transit, limiting their health benefits. Although phytosomes are widely prepared for stabilization of phenolics, their stability within food matrices remains underexplored. This study evaluated the ability of a food‐grade phytosome to improve the stability of bioactive phenolics from a *Cyclopia subternata* extract (CSE). Free and phytosome‐encapsulated extracts (CSE‐P) were incorporated into model beverages at pH 3, 5, and 7 and assessed during accelerated storage (40°C/90 days), simulated gastrointestinal digestion (INFOGEST 2.0), and simulated passive intestinal membrane diffusion (cellulose dialysis tubing). Phenolic degradation during storage was strongly pH‐dependent, with mangiferin, isomangiferin, and 3′,5′‐di‐β‐d‐glucopyranosylphloretin showing 80%–100% degradation within 10 days at pH 7, whereas 3‐β‐d‐glucopyranosyliriflophenone was the most stable compound across all experiments. Phytosomal encapsulation improved phenolic stability at all pH levels. Encapsulation efficiency and loading capacity decreased during storage, reaching near‐zero values, indicating phytosome destabilization. During simulated digestion, phenolics remained stable during the gastric phase, while partial degradation of mangiferin, isomangiferin, and scolymoside occurred during the intestinal phase in the presence of digestive enzymes. Bioaccessibility was comparable between CSE (70%–104%) and CSE‐P (67%–99%). Phytosomal encapsulation significantly reduced phenolic passive membrane diffusion, suggesting prolonged release from the phospholipid matrix. These findings demonstrate that phytosomes can improve the storage stability of CSE phenolics in acidified beverages while modulating phenolic behavior during gastrointestinal transit.

**Practical Applications:**

Phenolic stability in phytosomes has been minimally investigated in food matrices; these findings provide valuable knowledge to support their application in food products.

**Abbreviations**
CSE: *Cyclopia subternata* extract
CSE‐P: *Cyclopia subternata* extract‐loaded phytosomes
EE: encapsulation efficiency
HPLC: high‐performance liquid chromatography
IDG: 3‐β‐d‐glucopyranosyl‐4‐*O*‐β‐d‐glucopyranosyliriflophenone
LC: loading capacity
PC: phosphatidylcholine
PDG: 3′,5′‐di‐β‐d‐glucopyranosylphloretin
PdI: polydispersity index
SGF: simulated gastric fluid
SIF: simulated intestinal fluid

## 1 Introduction

The concentration of phenolic compounds in functional foods and beverages before and after ingestion is critical, as their health benefits are often dose‐dependent (Tian et al. 2025). However, the stability and biological availability of these phenolic compounds are affected by the presence of other macro‐ and micronutrients, as well as environmental factors (Gupta et al. 2023). These factors are relevant during both shelf storage and gastrointestinal transit, highlighting the need to improve phenolic stability and their availability in food systems.

Lipid‐based delivery systems, particularly nano‐phytosomes composed of phenolic–phospholipid complexes, have emerged as promising strategies to protect phenolic compounds from unfavorable environments (Chaudhary and Rajora 2025). Although phytosomes are increasingly being prepared for incorporation into functional foods and beverages, research focuses on immediate functionality and preclinical performance (Ghanbarzadeh et al. 2016). In contrast, shelf‐life stability in relevant food matrices remains underexplored. Limited studies have evaluated the stability of phenolic‐based phytosomes in food systems such as model juice solutions, acidic beverages, mayonnaise, and yoghurt containing rutin (Babazadeh et al. 2017), *Echinacea* extract (Molaveisi et al. 2021), resveratrol (Rabbani et al. 2021), and *Aucklandia costus* bioactives (Abdulqahar et al. 2022), respectively. Collectively, these studies demonstrated improved phenolic stability after phytosomal encapsulation, high physical stability of the phytosomes, and minimal effects on product quality attributes.

The gastrointestinal stability of phytosomes is also important, as it determines the concentration of phenolic compounds reaching the small intestine. Following ingestion, phytosomes are exposed to digestive enzymes and varying pH conditions throughout the upper gastrointestinal tract (Verhoeckx et al. 2015). Studies have reported improved phenolic stability during digestion following phytosomal encapsulation. However, many investigations assess only total phenolic content estimated using the Folin–Ciocalteu assay and/or antioxidant activity (Morales et al. 2025), often without direct comparison to free phenolic extracts or compounds. Since the stability of total phenolic content and antioxidant activity does not necessarily correlate with the stability of individual phenolics, high‐performance liquid chromatography (HPLC)‐based quantification of quantifiable phenolic compounds provides a more specific and sensitive assessment (Sik et al. 2022) to evaluate the extent to which individual phenolic compounds from different classes are affected by gastrointestinal conditions.

In addition to stability, gastrointestinal absorption of phenolics is a major determinant of their activity. Typically, only 5%–10% of phenolic compounds are absorbed in their native forms in the small intestine via passive diffusion (Ávila‐Román et al. 2021). Poor and slow diffusion through intestinal membranes is associated with their unfavorable physicochemical properties, including high molecular weight, multiple phenolic rings, and large polar surface area (Lipinski et al. 2001). Phytosomes, which have been associated with controlled release of phenolic compounds, can possibly mitigate this by preventing excessive local concentration of phenolic compounds in the small intestine and providing large molecular weight, slow‐diffusing compounds with more time to cross the intestinal barrier, thereby enhancing bioavailability and maximizing potential health benefits (Koppula et al. 2025).

The present study investigates a previously developed food‐grade phytosomal formulation encapsulating bioactive and labile phenolic compounds from a *Cyclopia subternata* extract (CSE) (Human, Aucamp, et al. 2023). The free and phytosome‐encapsulated phenolics will be evaluated in terms of stability during shelf storage in model beverage solutions, stability during simulated gastrointestinal digestion, and passive membrane diffusion in a simulated gastrointestinal model to establish a product‐centered understanding of the fate of these phenolics before reaching their site of action.

## 2 Materials and Methods

### 2.1 Chemicals

Lipoid S 45 (PC, a fat‐free soybean lecithin containing 45% phosphatidylcholine) was obtained from Brenntag (Johannesburg, Gauteng, South Africa). Pepsin from porcine gastric mucosa (CAS #: 1003626739), porcine pancreatin (CAS #: 1003540250), porcine bile extract (CAS #: 1001662553), hemoglobin from bovine blood (CAS #: 1002492802), dialysis tubing (D0405, flat width 23 mm, molecular weight cutoff 14,000, 99.99% retention), dialysis tubing closures, and all other analytical‐grade and HPLC‐grade chemicals and solvents were obtained from Merck (Darmstadt, Germany). Authentic reference standards for the quantification of phenolic compounds were obtained from Phytolab (Vestenbergsgreuth, Germany; mangiferin and 3‐β‐d‐glucopyranosyliriflophenone), Extrasynthese (Genay, France; luteolin and eriocitrin), and Merck (hesperidin). Deionized and HPLC‐grade water were prepared using an Elix water purification system and a Milli‐Q Academic water purification system, respectively, from Merck.

### 2.2 Preparation of Green CSE and CSE‐Loaded Phytosomes (CSE‐P)

CSE was prepared as described in detail by Human et al. (2021). Briefly, extracts were prepared from shredded, dried, and milled *C. subternata* shoots using 40% aqueous ethanol in a 1:10 ratio (m/v) for 30 min. Ethanol was removed from the extracts using a rotary evaporator under vacuum at 40°C (Rotavap, Büchi Labortechnik AG, Flawil, Switzerland), followed by freeze‐drying (Virtis Advantage Plus freeze‐drier, SP Scientific, Warminster, PA, USA) and storage in a desiccator until use.

CSE‐P was prepared using a modified version of the optimized procedure by Human, Aucamp, et al. (2023) as described in Tobin et al. (2026). Briefly, CSE and PC were dissolved (4.9:1, PC:CSE m/m ratio) in 90% (v/v) aqueous ethanol (2.4 mg/mL solids) by sonication followed by stirring at 40°C for 2 h. Ethanol was removed by rotary evaporation. The residue was suspended in deionized water (12 mg/mL solids) and subjected to mechanical shearing (10,000 rpm/10 min) using a benchtop disperser (T25 digital Ultra‐Turrax, IKA, Staufen, Germany). Freeze‐dried CSE‐P was stored in a desiccator at room temperature until further use.

### 2.3 Preparation of CSE and CSE‐P Model Beverage Solutions

Model beverage solutions in deionized water (pH 4.6) and in buffer solutions (pH 3, 5, and 7) containing CSE (2 g/L) and CSE‐P (16 g/L) were prepared. Buffers were prepared by dissolving NaH_2_PO_4_.2H_2_O in deionized water (0.09 M) and adjusting the pH using either 2 M KOH or 6 M HCl. Solutions were prepared in triplicate for the shelf‐life and quadruplicate for the simulated digestion and passive simulated gastrointestinal membrane diffusion experiments. The concentration of CSE was based on the amount of soluble solids present in a cup of *C. subternata* herbal tea, corresponding to the dosage previously shown to exhibit antiallergic activity (Yoshida et al. 2020). The concentration used for CSE‐P was adjusted to be equivalent to that of CSE, based on the phenolic compounds (%) in CSE‐P.

### 2.4 Shelf‐Life Storage

An aliquot (10 mL) of each model solution at pH 3, 5, and 7, prepared in triplicate, with either CSE or CSE‐P, was transferred to 20‐mL amber glass vials and sealed with screw caps. Sealed vials were stored in a laboratory oven (Labex, Johannesburg, Gauteng, South Africa) at 40°C/90 days. Accelerated storage conditions were selected based on the ICH guidelines for formulations that contain bioactive constituents (EMA 2006). Triplicate vials for each sample were removed at predetermined time points (*n* = 7), including the initial time point at day 0. Aliquots of the sampled solutions were stored at −20°C until further analysis. Before HPLC analysis, 10% (v/v) dimethyl sulfoxide was added to CSE‐P samples, followed by 5‐min sonication (Bransonic 12, Branson, Danbury, CT, USA; 80 W, 50/60 Hz) to disrupt the phytosomal structures.

### 2.5 Simulated In Vitro Digestion

#### 2.5.1 Enzyme Activity and Bile Acid Assays

Pepsin and trypsin (in pancreatin) activity assays were completed using a microplate reader (Synergy HT, Biotek, Agilent, Santa Clara, CA, USA) to calculate the volumes of enzyme solutions required as described in detail in the standardized INFOGEST 2.0 procedure. No modifications were made, except that measurements were made in flat‐bottom, 96‐well microplates instead of cuvettes. The bile extract was analyzed using a bile acid assay test kit (MAK309, Merck) to determine the bile acid content (mmol/g).

#### 2.5.2 Preparation of Solutions

The preparation of simulated gastric fluid (SGF) and simulated intestinal fluid (SIF) was based on Brodkorb et al. (2019) and is shown in Table S1. NaHCO_3_ was replaced with NaCl at the same molarity to prevent pH drift during gastric digestion due to CO_2_ release, as suggested by Brodkorb et al. (2019). CaCl_2_ (0.3 M) was added to an aliquot of these solutions before use to obtain a final concentration of 0.15 and 0.6 mM in the SGF and SIF, respectively. Bile extract solution (20 mg/mL) was prepared by dissolving in heated SIF by sonication and kept warm until use. Pepsin (10 mg/mL) was prepared by dissolving in water by vortexing and kept on ice until further use. Pancreatin (10 mg/mL) was prepared in preheated SIF 30 min before use.

#### 2.5.3 Digestion

Digestion for each sample was performed in quadruplicate on separate days in the presence and absence of enzyme and bile extract solutions. This was done in a Thermomixer (Eppendorf, Hamburg, Germany), at 37°C at 300 rpm in a 24‐well deep‐well plate. Samples included CSE and CSE‐P in deionized water. An example of the volumes and final concentrations of components added during the digestion procedure is shown in Table S2. Before gastric digestion, samples (2 mL) were taken of the freshly prepared CSE and CSE‐P.

Gastric digestion entailed the mixing and incubation of the sample solution (2 mL) with SGF (1.6 mL), pepsin (0.07 mL), and HPLC‐grade water to make up the volume to 4 mL. For each sample, a well containing all ingredients, excluding the enzyme solution, was also prepared, and HPLC‐grade water was used to fill the sample to a final volume of 4 mL. The mixtures was adjusted to pH 3 using a predetermined volume of 0.1 M hydrochloric acid, which differed depending on the sample. After 2 h of incubation, a sample (2 mL) was taken from each of the gastric wells.

The digestion phases were sequential; the intestinal phase entailed the addition of SIF (1.6 mL), pancreatin (0.497 mL), and bile solution (1.427 mL) to gastric phase wells that were not sampled. For each sample, a well containing all ingredients with an additional 1.9 mL of SIF and excluding the enzyme and bile solutions was also prepared. HPLC‐grade water was used to fill the wells to a final volume of 8 mL. The mixtures was adjusted to pH 7 using a predetermined volume of 0.1 M NaOH, which differed depending on the sample. After 2 h of incubation, a sample (2 mL) was taken from each of the intestinal wells.

#### 2.5.4 Sample Preparation for HPLC

All samples taken were immediately mixed with 200 µL ascorbic acid (100 mg/mL) and ice‐cold acetonitrile (6 mL). The mixtures were centrifuged at 4°C for 5 min at 13,520 × *g* (Universal 320 R, Hettich, Tuttlingen, Germany). A portion of each supernatant (2 mL) was dried in a SpeedVac concentrator (Savant SPD2010, Thermo Fisher Scientific, Waltham, MA, USA) for 7 h at 45°C, vacuum pressure of 5.1, and ramp 4. The dried sample was reconstituted with 500 µL of 10% (v/v) dimethyl sulfoxide, sonicated for 10 min, and frozen until analysis.

### 2.6 Simulated Passive Gastrointestinal Membrane Diffusion

#### 2.6.1 Preparation

Dialysis tubing was prepared by soaking in deionized water overnight at room temperature. SGF and SIF were prepared as described in Section 2.5.2. Ascorbic acid (ca. 1 mg/mL final concentration) was added to the samples, SGF and SIF, to prevent phenolic degradation during experiments.

#### 2.6.2 Experiment

The SGF and SIF (200 mL) were placed in jacketed vessels (receiver compartment) connected to a circulating water bath at 37°C and magnetically stirred at 100 rpm (Lasec, Cape Town, Western Cape, South Africa). The samples (10 mL CSE or CSE‐P in deionized water) were placed in the dialysis tubing (donor compartment) and sealed with dialysis tubing closures. The volume ratio of the donor to the receiver compartment was 1:20 (v/v). The solution in the receiver compartment was sampled at 0, 0.25, 0.5, 1, 2, 3, 5, and 7 h. Sink conditions were maintained by replacing the solution removed from the receiver compartment with an equal volume of preheated SGF or SIF after each sampling. Initial CSE‐P (*t* = 0 h) samples were prepared for HPLC by taking 2 mL of sample, adding the same volume of HPLC water and 400 µL of dimethyl sulfoxide (final concentration = 10%), then sonicating. The initial CSE samples were analyzed as is.

### 2.7 Quantification of Phenolic Compounds

Ascorbic acid (ca. 1 mg/mL final concentration) was added to all CSE and CSE‐P samples before filtering using 0.22‐µm pore‐size Millex‐HV syringe filters (Merck). The phenolic content of these samples was determined using a validated HPLC method for *C. subternata* (De Beer et al. 2012). Analysis was performed on an Agilent 1200 HPLC system (Agilent) equipped with a diode array detector. Gradient separation was achieved on a Gemini‐NX C_18_ (150 × 3 mm; 3 µm; 110 Å) column (Phenomenex, Torrance, CA, USA) at a flow rate of 0.43 mL/min and a column temperature of 30°C with aqueous acetic acid (2% v/v) and acetonitrile as mobile phases. Compounds were quantified at 288 and 320 nm. Nine‐point calibration curves with authentic reference standards were used for quantification. Where authentic reference standards were not available, response factors were used as specified in Human et al. (2021).

### 2.8 Phytosome Quality Parameters

The phytosome quality parameters were measured for CSE‐P in model solutions in deionized water (control) and after shelf‐life storage in pH 3, 5, and 7 solutions.

The encapsulation efficiency (EE) and loading capacity (LC) were determined by separating the bound phenolics from the unbound phenolics, based on differences in solubility, as previously detailed in Human, Aucamp, et al. (2023), with no modifications. The EE and LC were calculated based on the sum of the quantified phenolic compounds using the equations given in Tobin et al. (2026). The sum of quantified compounds included 3‐β‐d‐glucopyranosyl‐4‐*O*‐β‐d‐glucopyranosyliriflophenone (IDG), 3′,5′‐di‐β‐d‐glucopyranosylphloretin (PDG), eriocitrin, hesperidin, mangiferin, isomangiferin, and scolymoside.

The vesicle size, polydispersity index (PdI), and zeta potential were determined using dynamic light scattering (Zetasizer Nano Series 2590, Malvern Panalytical, Malvern, UK) after dilution of samples to 1 mg/mL total solids using deionized water. Analysis was performed on the same day as the experiments.

### 2.9 Statistical Analysis

Univariate analysis of variance (ANOVA) of regression parameters for kinetic modeling (first‐order degradation rate constants and second‐order passive membrane diffusion rate constants) and the quantitative data, including the phytosome quality parameters, compound concentrations before and after in vitro digestion, as well as the % passive diffusion of quantified compounds into the receiver compartment for the simulated gastrointestinal membrane diffusion experiment, was done according to the experimental design of each experiment. The normality of residuals was evaluated using the Shapiro–Wilk test. A probability level of 5% was considered significant. Fisher's LSD was calculated at the 5% level to compare means for significant effects. ANOVAs were performed using SAS software (Version 9.4, SAS Institute Inc., Cary, CA, USA).

#### 2.9.1 Shelf Life—Kinetic Modeling

Concentrations of individual phenolic compounds in CSE and CSE‐P in the model solutions (pH 3, 5, and 7) were modeled using nonlinear regression with time as the independent variable. Zero‐, first‐, and second‐order models were fitted for each replicate (*n* = 3) using the NLIN procedure of SAS software (Version 9.4; SAS Institute Inc.). The models were evaluated using the adjusted coefficient of determination (*R* ^2^_adj_). Although the first‐order model (Equation 1) did not give the best fit for all compounds and conditions, it was selected to predict compound degradation as in most cases, it provided moderate to good fits (*R* ^2^_adj_ > 0.75).

(1) $$C=C_{0}\left(\exp\left(-kt\right)\right),$$

where *C* is the concentration of the phenolic compounds (g/100 g extract) after time *t* (days), *C*_0_ is the initial concentration (g/100 g extract) at *t* = 0 days, and *k* is the reaction rate constant.

#### 2.9.2 Simulated In Vitro Digestion and Passive Gastrointestinal Membrane Diffusion

Four independently replicated experiments were conducted for each solution type (i.e., CSE and CSE‐P in water) for the in vitro simulated digestion experiment (including gastric and intestinal phases with and without enzymes/bile) using a completely randomized experimental design. The simulated passive gastrointestinal membrane diffusion experiment was performed similarly, but only in the absence of enzymes and bile.

For the in vitro digestion, the bioaccessibility for each phenolic compound was calculated using Equation (2):

(2) $$\mathrm{Bioaccessibility}\;\left(\%\right)=\frac{\left[\mathrm{intestinal}\;\mathrm{phase}\;\mathrm{with}\;\mathrm{enzyme}\right]\;}{\left[\mathrm{control}\right]}\times 100,$$

where [intestinal phase with enzyme] is the concentration of the phenolic compound (g/100 g extract) in the supernatant obtained after protein precipitation and centrifugation (refer to Section 2.5.4) of the intestinal phase in the presence of enzymes and bile, and [control] is the concentration of the phenolic compound (g/100 g extract) before digestion in CSE or CSE‐P.

For the simulated passive gastrointestinal membrane diffusion experiment, the % passive diffusion for each phenolic compound into the receiver compartment was calculated using Equation (3):

(3) $$\mathrm{Passive}\;\mathrm{diffusion}\;\left(\%\right)=\left(\frac{X}{X_{i}}\right)\times 100,$$

where *X* is the cumulative phenolic compound content (mg/mL) in the receiver compartment at a given time point, and *X_i_* is the initial phenolic compound content (mg/mL) in the donor compartment.

To determine the passive membrane diffusion rate of compounds through the dialysis membrane, empirical kinetic models were fitted to the data using nonlinear regression with time as an independent variable using the NLIN procedure of SAS software. First‐ and second‐order models were fitted to the data for each experimental replicate (*n* = 4). The second‐order model (Equation 4) gave the best fit (based on the adjusted coefficient of correlation [*R* ^2^_adj_]) and was used to describe the passive membrane diffusion kinetics of quantified phenolic compounds.

(4) $$C=C_{0}-\frac{C_{0}}{\left(1+C_{0}kt\right)},$$

where *C* is the diffusion of the compound into the receiver compartment (%) at time *t* (h), *C*_0_ is the initial concentration (100 %) at *t* = 0 h, and *k* is the passive membrane diffusion rate constant.

## 3 Results

### 3.1 Shelf Life

#### 3.1.1 Phenolic Stability

Based on the *R* ^2^_adj_ values, the first‐order model had a moderate to good fit for all compounds and samples (*R* ^2^_adj_ > 0.75; Table S3) and was used to determine reaction rate constants for comparison of samples and solution pH (Table 1). Mangiferin, isomangiferin, PDG, and eriocitrin in pH 7 model solutions were 80%–100% degraded at the first time point (10 days) (Figure 1). Thus, reaction rate constants could not be determined for these samples, as only two to three nonzero time points were available.

**TABLE 1:** First‐order^a^ reaction rate constants (*k*, mean ± SD, *n* = 3) for quantified phenolic compounds in *Cyclopia subternata* extract (CSE) and CSE‐loaded phytosomes (CSE‐P) in model solutions (pH 3, 5, and 7) stored at 40°C for 90 days.

		*k* (day^−1^ × 10^3^)						
Sample	pH	IDG	Eriocitrin	PDG	Hesperidin	Mangiferin	Isomangiferin	Scolymoside
CSE	3	4.85c ± 0.18	6.50b ± 1.17	6.33c ± 0.43	8.41c ± 0.47	38.34c ± 1.43	9.53c ± 0.38	10.18d ± 0.54
	5	1.71d ± 0.14	12.36a ± 0.98	24.57a ± 3.01	7.04c ± 1.18	99.85a ± 5.06	34.43a ± 0.69	15.89c ± 2.46
	7	21.69a ± 2.31	nd	nd	68.08a ± 0.83	nd	nd	105.71a ± 4.89
CSE‐P	3	1.59d ± 0.28	4.66c ± 0.22	4.75c ± 1.21	2.81d ± 0.13	25.80d ± 0.42	8.53c ± 0.05	4.84e ± 0.33
	5	1.00d ± 0.16	5.77bc ± 0.34	11.61b ± 0.29	2.37d ± 0.30	62.23b ± 0.87	22.57b ± 0.76	5.63de ± 0.66
	7	18.27b ± 1.82	nd	nd	37.27b ± 1.58	nd	nd	58.32b ± 4.39

*Note*: Different letters allocated to *k* values within a compound indicate significant differences (*p* < 0.05).

Abbreviations: IDG, 3‐β‐d‐glucopyranosyl‐4‐*O*‐β‐d‐glucopyranosyliriflophenone; nd, not determined; PDG, 3′,5′‐di‐β‐d‐glucopyranosylphloretin.

^a^ First‐order model: $C=C_{0}\left(\exp\left(-kt\right)\right)$, where *C* is the concentration of the compound (g/100 g extract), *C*_0_ is the initial concentration of the compound (g/100 g extract), *t* is the time (days), and *k* is the reaction rate constant.

**FIGURE 1:**
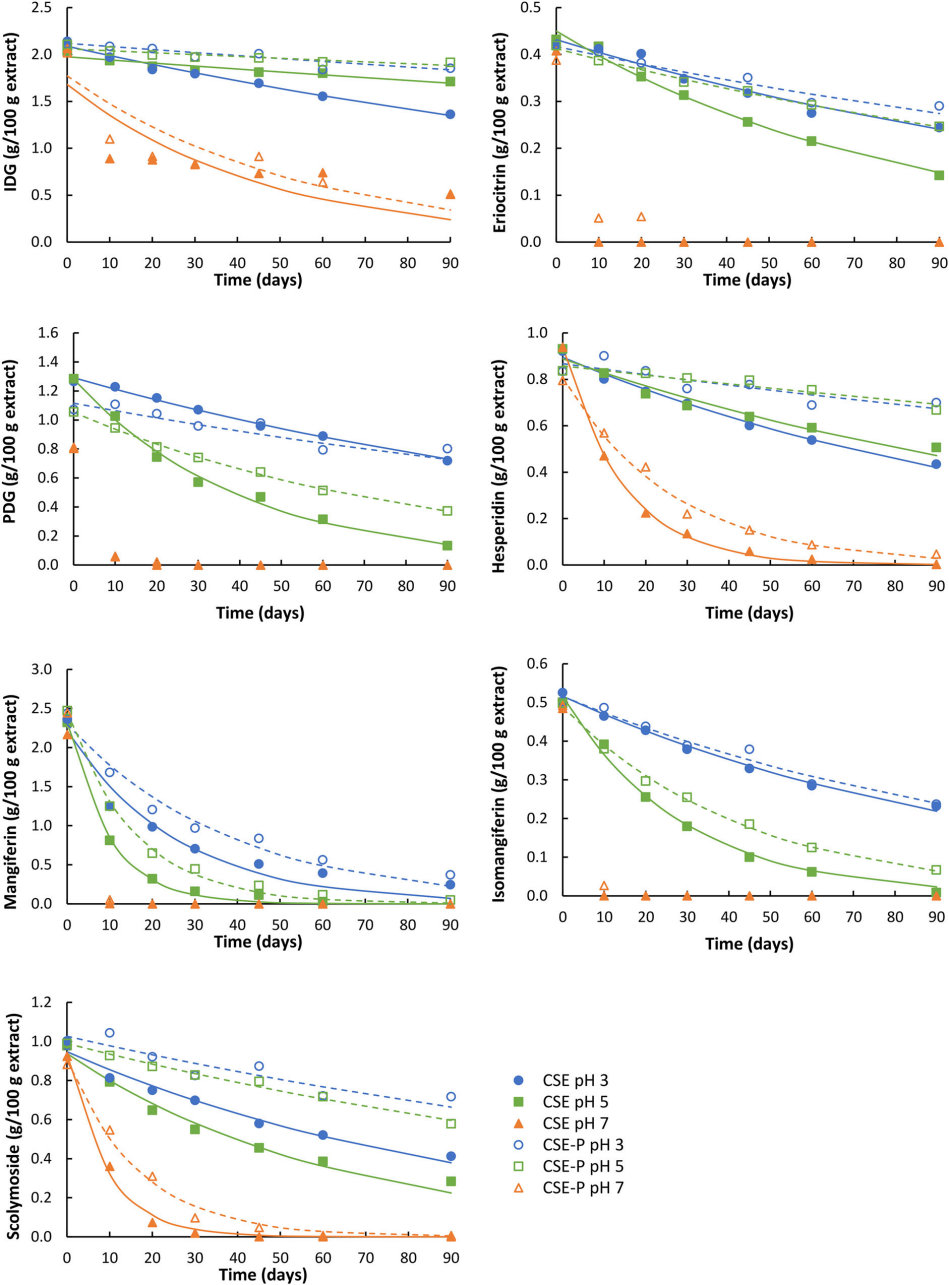
Experimental and predicted content (g/100 g extract; mean, *n* = 3) of quantified compounds in *Cyclopia subternata* extract (CSE) and CSE‐loaded phytosomes (CSE‐P) in model solutions during storage at 40°C at pH 3, 5, and 7. Experimental data are indicated with markers. Predicted values are based on the first‐order model and are indicated by solid (CSE) and dashed (CSE‐P) lines. IDG; 3‐β‐d‐glucopyranosyl‐4‐*O*‐β‐d‐glucopyranosyliriflophenone; PDG, 3′,5′‐di‐β‐d‐glucopyranosylphloretin. Data for eriocitrin, PDG, mangiferin, and isomangiferin were not modeled at pH 7 due to complete degradation after about 10–20 days.

Differences were observed in the relative stability of the compounds for CSE and CSE‐P, irrespective of pH. Generally, mangiferin was the most unstable, as shown by its high reaction rate constants at pH 3 and 5 (25.80–38.34 and 62.23–99.85 × 10^−3^ day^−1^, respectively), as well as rapid degradation at pH 7, followed by isomangiferin. IDG was the most stable, as shown by the smallest reaction rate constants (1.00–21.69 × 10^−3^ day^−1^). The trends for the remaining compounds, which were considered to have intermediate stability, varied depending on the solution pH and sample.

Compounds degraded faster at pH 7, as shown by significantly (*p* < 0.05) higher rate constants (Table 1) and rapid degradation for compounds not modeled (Figure 1). For all the compounds, except IDG and hesperidin, smaller degradation rate constants were observed at pH 3 than at pH 5, albeit not always significant (*p* ≥ 0.05). The extent of the pH effect varied depending on the compound. For example, mangiferin in CSE degraded 2.6‐fold faster in the pH 5 model solution than in the pH 3 model solution, whereas for scolymoside in the same solutions, this was only 1.6‐fold.

Smaller reaction rate constants (significant in most cases [*p* < 0.05]) for phenolic degradation in CSE‐P compared to CSE were observed, indicating stability imparted by phytosomal formulation. The magnitude of this effect was also dependent on the compound and pH. For example, when comparing pH within a compound, IDG was 1.2‐fold and 3.1‐fold more stable in CSE‐P than CSE at pH 7 and pH 3, respectively. When comparing across compounds at the same pH, isomangiferin was 1.5‐fold more stable in CSE than in CSE‐P at pH 5, whereas PDG was 2.1‐fold more stable in CSE‐P than in CSE at the same pH.

#### 3.1.2 Phytosome Quality Parameters

Table 2 shows the phytosome quality parameters for CSE‐P in deionized water (control without storage) and the model beverage solutions (pH 3, 5, and 7) after 90 days of storage. The control was prepared in deionized water to observe the effect of pH and storage conditions holistically. The control had EE and LC values of 83.3% and 14.2%, respectively. Its vesicle size, PdI, and zeta potential were 469 nm, 0.66, and −18.65 mV, respectively. After storage, the EE and LC decreased significantly (*p* < 0.05) to below 17.3% and 0.03%, respectively. Even though major changes (increases and decreases) were observed in vesicle size and PdI, none were significantly (*p* ≥ 0.05) different from the control before storage. The absolute values of the zeta potential decreased significantly (*p* < 0.05) after storage for all model beverage solutions.

**TABLE 2:** Phytosome quality parameters (mean ± SD, *n* = 3) of *Cyclopia subternata* extract‐loaded phytosomes (CSE‐P) in model solutions before (control) and after 90 days of storage.

Model solution	Encapsulation efficiency (%)	Loading capacity (%)	Vesicle size (nm)	PdI	Zeta potential (mV)
Control^a^	83.4a ± 0.9	14.2a ± 0.14	469a ± 25	0.66a ± 0.04	−18.65b ± 8.65
pH 3	0.4d ± 0.1	0.00b ± 0.00	1030a ± 775	0.84a ± 0.22	−1.92a ± 0.64
pH 5	17.3b ± 5.3	0.03b ± 0.01	1343a ± 512	0.74a ± 0.43	−3.73a ± 2.96
pH 7	11.3c ± 1.3	0.02b ± 0.00	575a ± 35	0.70a ± 0.10	−3.33a ± 0.99

*Note*: Different letters within a quality parameter indicate significant differences (p < 0.05).

^a^ Control is in deionized water.

Abbreviation: PdI, polydispersity index.

### 3.2 Simulated In Vitro Digestion

Significant (*p* < 0.05) changes after digestion with enzymes for CSE were only noted for mangiferin, isomangiferin, and scolymoside after the intestinal phase (Table 3). The phenolic compounds in CSE were stable during both gastric digestion with and without enzymes/bile and intestinal digestion in the absence of enzymes/bile.

**TABLE 3:** Content (g/100 g extract) of quantified phenolic compounds (mean ± SD, *n* = 4) in *Cyclopia subternata* extract (CSE) and CSE‐loaded phytosomes (CSE‐P) before (control) and after simulated gastrointestinal digestion with and without enzymes.

Solution type	Presence of enzymes	Digestion phase	IDG	Eriocitrin	PDG	Hesperidin	Mangiferin	Isomangiferin	Scolymoside
CSE	—	Control	2.001ab ± 0.029	0.340a ± 0.009	1.168ab ± 0.034	0.926abc ± 0.018	2.317a ± 0.038	0.502a ± 0.099	1.093a ± 0.019
	—	Gastric	1.990ab ± 0.162	0.347a ± 0.034	1.180ab ± 0.086	0.928abc ± 0.064	2.318a ± 0.185	0.499a ± 0.040	1.101a ± 0.096
		Intestinal	2.118a ± 0.061	0.345a ± 0.013	1.225a ± 0.042	0.976a ± 0.038	2.428a ± 0.089	0.502a ± 0.021	1.109a ± 0.056
	+	Gastric	2.042ab ± 0.235	0.347a ± 0.034	1.207ab ± 0.123	0.923abc ± 0.099	2.336a ± 0.231	0.505a ± 0.051	1.114a ± 0.116
		Intestinal	2.079ab ± 0.186	0.335a ± 0.035	1.137ab ± 0.061	0.880abc ± 0.055	1.953b ± 0.138	0.428b ± 0.029	0.765c ± 0.061
		% Bioaccessibility^a^	103.9	98.6	97.3	95.0	84.3	85.3	70.0
CSE‐P	—	Control	2.018ab ± 0.050	0.346a ± 0.018	1.157ab ± 0.017	0.927abc ± 0.019	2.297a ± 0.019	0.506a ± 0.017	1.102a ± 0.034
	—	Gastric	2.033ab ± 0.231	0.343a ± 0.054	1.082bcd ± 0.155	0.923abc ± 0.117	2.230a ± 0.288	0.488a ±0.077	1.053ab ± 0.176
		Intestinal	2.125a ± 0.216	0.339a ± 0.038	1.095abc ± 0.090	0.930ab ± 0.085	2.221a ± 0.178	0.489a ± 0.043	1.082a ± 0.157
	+	Gastric	1.826b ± 0.255	0.304a ± 0.034	0.959d ± 0.103	0.818c ± 0.098	1.922b ± 0.158	0.414b ± 0.029	0.923b ± 0.123
		Intestinal	1.998ab ± 0.199	0.323a ± 0.019	0.971 cd ± 0.063	0.846bc ± 0.055	1.731b ± 0.094	0.410b ± 0.024	0.740c ± 0.034
		% Bioaccessibility^a^	99.0	93.4	84.0	91.3	75.4	80.9	67.2

*Note*: Values for a compound with different letters are significantly different (*p* < 0.05). A minus symbol (−) indicates the absence and a plus symbol (+) the presence of the respective enzymes.

Abbreviations: IDG, 3‐β‐d‐glucopyranosyl‐4‐*O*‐β‐d‐glucopyranosyliriflophenone; PDG, 3′,5′‐di‐β‐d‐glucopyranosylphloretin.

^a^ Bioaccessibility (%) = (Intestinal phase with enzyme × 100)/control.

No significant (*p* ≥ 0.05) differences were observed in the phenolic composition of CSE and CSE‐P control samples. Phenolic stability in CSE‐P solutions, without enzymes, displayed the same pattern as the equivalent CSE solutions. Mangiferin and isomangiferin in CSE‐P significantly decreased (*p* < 0.05) in the gastric phase with enzymes, in addition to the intestinal phase, which was not observed for CSE.

The relative bioaccessibility of the compounds from CSE was as follows: scolymoside < mangiferin < isomangiferin < hesperidin < PDG < eriocitrin < IDG (Table 3). The relative stability for CSE‐P was similar, except that PDG was more unstable than hesperidin. In most cases, the bioaccessibility (%) of a compound was also similar for CSE‐P and CSE (67%–99% and 70%–104%, respectively).

### 3.3 Simulated Passive Gastrointestinal Membrane Diffusion

The passive diffusion (%) of the phenolic compounds from the donor to the receiver compartment increased significantly (*p* < 0.05) over time for all quantified compounds (Figure 2; Table S4). Notable trends could be observed: The phenolic compound diffusion (%) appeared to be (1) more rapid and reached higher levels in the gastric phase than the intestinal phase and (2) less rapid for CSE‐P compared to CSE.

**FIGURE 2:**
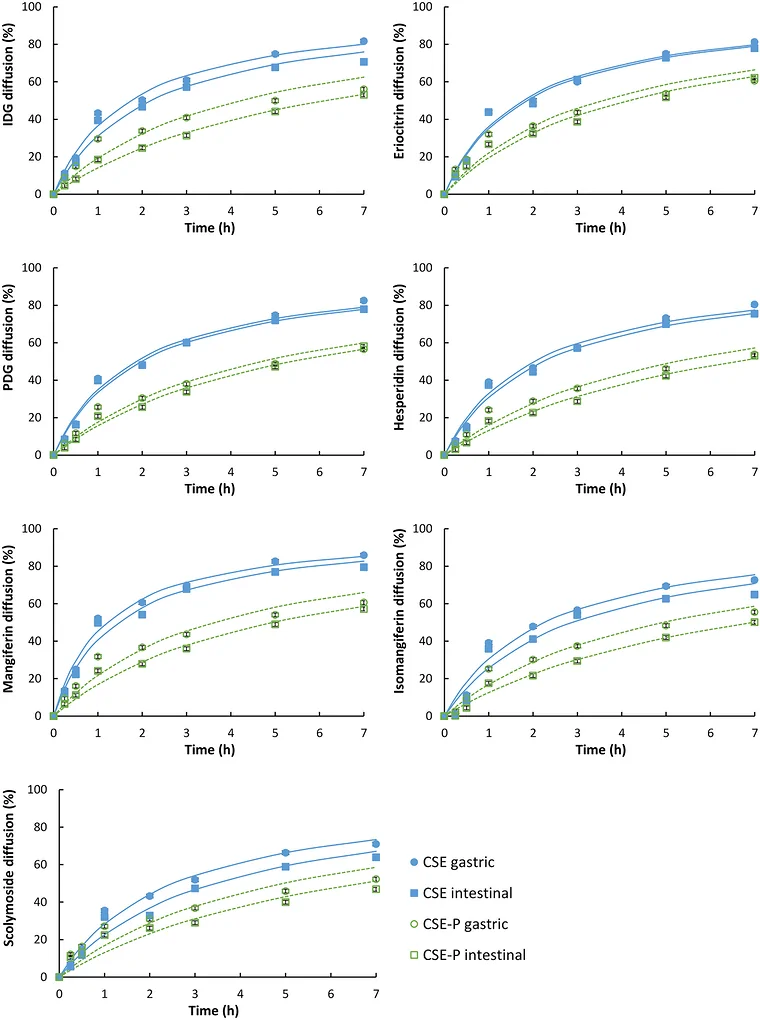
Experimental and predicted diffusion (%; mean) of quantified compounds in *Cyclopia subternata* extract (CSE) and CSE‐loaded phytosomes (CSE‐P) into the receiver compartment containing simulated gastric and intestinal fluids (without enzymes) during simulated passive gastrointestinal membrane diffusion using CSE and CSE‐P. Predicted values are based on the second‐order model and are indicated by solid (CSE) and dashed (CSE‐P) lines. IDG; 3‐β‐d‐glucopyranosyl‐4‐*O*‐β‐d‐glucopyranosyliriflophenone; PDG, 3′,5′‐di‐β‐d‐glucopyranosylphloretin.

Empirical kinetic models were fitted to diffusion data (%). Based on the *R* ^2^_adj_ (>0.9), the second‐order model gave the best fit (Table S5; Figure 2) and was used to predict the passive membrane diffusion rate. These rate constants for phenolic compounds in CSE and CSE‐P were between 2.92‐8.31 × 10^3^ [(% diffusion)^−1^ h^−1^] and between 1.44–2.78 × 10^3^ [(% diffusion)^−1^ h^−1^], respectively, across the two digestion phases (Table 4).

**TABLE 4:** Second‐order^a^ passive diffusion rate constants (*k*, mean ± SD, *n* = 4) for phenolic compounds into the receiver compartment containing simulated gastric and intestinal digestive fluids (without enzymes) during simulated passive gastrointestinal diffusion using *Cyclopia subternata* extract (CSE) and CSE‐loaded phytosomes (CSE‐P).

Compound	Passive diffusion rate constants [*k*, (%diffusion^−1^ h^−1^) × 10^3^]			
	CSE		CSE‐P	
	Gastric	Intestinal	Gastric	Intestinal
IDG	5.75a ± 0.41	4.52b ± 0.47	2.38c ± 0.50	1.64d ± 0.08
Eriocitrin	5.65a ± 0.22	5.39a ± 0.19	2.42b ± 0.40	2.42b ± 0.16
PDG	5.38a ± 0.44	5.03a ± 0.30	2.14b ± 0.46	1.86b ± 0.19
Hesperidin	4.92a ± 0.29	4.46b ± 0.16	1.91c ± 0.34	1.52d ± 0.10
Mangiferin	8.31a ± 0.77	6.84b ± 0.39	2.78c ± 0.36	2.02d ± 0.14
Isomangiferin	4.40a ± 0.60	3.46b ± 0.25	2.03c ± 0.20	1.44d ± 0.06
Scolymoside	3.95a ± 0.46	2.92b ± 0.40	2.02c ± 0.17	1.50d ± 0.17
Total phenolic content^b^	5.62a ± 0.45	4.71b ± 0.27	2.33c ± 0.37	1.78d ± 0.10

*Note*: Values for *k* for a compound with different letters are significantly different (*p* < 0.05).

Abbreviations: IDG, 3‐β‐d‐glucopyranosyl‐4‐*O*‐β‐d‐glucopyranosyliriflophenone; PDG, 3′,5′‐di‐β‐d‐glucopyranosylphloretin.

^a^ Second‐order model: $C=C_{0}-\left(C_{0}/\left(1+C_{0}kt\right)\right)$, where *C* is the diffusion of the compound into the receiver compartment at time *t* (hours), *C*_0_ is the initial concentration (100 %) at *t* = 0 h, and *k* is the diffusion rate constant.

^b^ Sum of quantified phenolic compounds.

For most compounds in CSE and CSE‐P, significantly (*p* < 0.05) higher diffusion rates were observed in the gastric phase. The relative diffusion rates also varied with compound and phase (Table 4). Scolymoside had the lowest rate constants for both phases for CSE, while hesperidin and isomangiferin had the lowest rate constants in the gastric and intestinal phases for CSE‐P, respectively. Mangiferin had the highest diffusion rate for CSE in both digestion phases and the gastric phase for CSE‐P, while eriocitrin had the highest diffusion rate for the intestinal phase in CSE‐P.

When comparing the diffusion rate constants for CSE and CSE‐P, rates for CSE‐P during both phases were significantly (*p* < 0.05) lower than those for CSE for all of the compounds for the respective phases. This was corroborated by the diffusion (%) to the receiver compartments after 7 h, which ranged between 64.3% and 85.9% and between 44.7% and 62.1% for compounds in CSE and CSE‐P, respectively, for both phases (Table S4).

## 4 Discussion

The health‐promoting effects of CSE phenolic compounds can only be realized if the appropriate concentration of bioactive compounds is available at the point of consumption and after ingestion. The phenolic compounds in CSE, with known antiallergic activity (Yoshida et al. 2020), are labile during shelf storage, as previously shown in model beverage solutions (Human, De Beer, et al. 2023), prompting their phytosomal formulation using food‐grade, cost‐effective constituents. The positive effect of phytosomal formulation on the stability of these phenolic compounds during processing (thermal and cold plasma) of model beverage solutions has already been shown (Tobin et al. 2026). The present study, therefore, evaluated the stability of CSE phenolics and their phytosomal formulations during shelf storage and simulated gastrointestinal digestion to determine the extent to which the original phenolic compounds would remain available for absorption in the small intestine.

### 4.1 Shelf‐Life Stability

Ready‐to‐drink (RTD) beverages typically have a long shelf life (>3 months). Therefore, stability was evaluated under accelerated conditions (40°C for 3 months) according to ICH guidelines (EMA 2006). Empirical kinetic modeling was used to determine phenolic degradation rate constants and compare treatments. Phytosome structural integrity was also assessed as an indicator of formulation stability.

The relative stability of the phenolic compounds was consistent with previous observations for CSE stored in model solutions with and without citric and ascorbic acid at 40°C for 90 days (Human, De Beer, et al. 2023). Mangiferin showed greater instability than its regioisomer, isomangiferin, in agreement with earlier studies (Beelders et al. 2015, 2017), likely related to glycosylation position (Beelders et al. 2017). The high stability of IDG may be explained by its chemical structure: the 4‐hydroxy group is particularly susceptible to oxygen‐radical formation, but 4‐*O*‐glycosylation limits this through steric hindrance (Beelders et al. 2017).

Phenolic stability was strongly pH‐dependent, with degradation increasing at higher pH values. Neutral pH conditions are known to accelerate phenolic oxidation, although the extent depends on compound structure (Friedman and Jürgens 2000). Beverage pH is important for both the quality and safety of RTD products (Aadil et al. 2019) and varies across beverage types. Acidified iced teas and juices generally have pH values of 3–4, while natural iced teas and juices are around pH 5 (Reddy et al. 2016) and dairy‐based beverages have a pH of approximately 7 (O'Connel and Fox 1999).

Higher degradation rates in CSE solutions than in CSE‐P solutions indicate that the phytosomal formulation, which can be due to the presence of phospholipids or complexation, protected the phenolic compounds in the model beverage matrices. Improved stability in phytosomal systems is widely attributed to hydrogen bonding between phenolic hydroxyl groups and the polar head groups of phospholipids, forming a phyto–phospholipid complex (Chaudhary and Rajora 2025). The presence of phospholipids could also possibly offer protection; however, this is not supported by the literature. However, due to the lack of a control sample containing a mixture of the components, the protective effect cannot be definitively linked to the phytosomal structure or presence of the phospholipids. While stabilization effects have been extensively documented, evidence demonstrating these effects in a food matrix remains limited to beverages, yoghurt, and mayonnaise (Abdulqahar et al. 2022; Babazadeh et al. 2017; Molaveisi et al. 2021; Rabbani et al. 2021), among others. In a model acidic beverage (pH 3.5) stored at 25°C/30 days, chicoric and chlorogenic acids degraded 1.4‐fold faster in free *Echinacea* extract than in the corresponding phytosomes (Molaveisi et al. 2021). This is in line with the 1.1‐ to 3.1‐fold faster phenolic degradation in CSE than CSE‐P at pH 3 observed in the current study. A resveratrol‐loaded phytosome exhibited 1.35‐fold higher antioxidant activity than the free compound in mayonnaise stored at 4°C for 60 days, although the observed difference was not statistically significant. The remaining studies investigating phytosomes in different beverages and in yoghurt did not report results for phenolic stability in their respective food matrices.

Phytosome structural stability is equally important, as it may predict the ability of the formulation to protect phenolics during storage. The CSE phytosomes were unstable, with almost no phenolic compounds remaining encapsulated after 90 days, an apparent increase in vesicle size (although not significant), and a substantial decrease in absolute zeta potential, irrespective of beverage pH. The drastic decrease in the encapsulated phenolic compounds in the phytosomes after completion of the storage period could be attributed to the release of the compounds, as the compounds are hydrophilic in nature and would have high affinity for the surrounding aqueous medium. The release of the phenolic compounds would likely follow a sustained release profile, widely associated with phytosomes (Koppula et al. 2025). Consequently, the protective effects of the phytosomes would likely gradually decrease as the storage period progresses and more phenolic compounds are released and lost after prolonged storage. In conjunction with the natural release profile of the phytosomes, the probable oxidation and hydrolysis of the phospholipid during storage (Cui and Decker 2016) could also result in collapse of the phytosomal structure and release of the phenolic compounds. Phospholipids have been shown to be more susceptible to oxidation in acidic environments (Vareltzis and Hultin 2007), which would explain the more drastic decrease in EE and LC at pH 3. Changes in the phytosome size could influence stability, gravitational separation, solubility, and bioavailability. The low absolute zeta potential indicates that the dispersions are unstable and more prone to coagulation and aggregation (Rabbani et al. 2021) after storage. Babazadeh et al. (2017) reported minor changes in particle size for rutin‐loaded phytosomes stored in milk, orange juice, and apple juice model beverages. However, the samples were only stored for 30 days. The EE and zeta potential of the rutin‐loaded phytosomes were not reported, but EE in the milk model solution was reduced significantly after sterilization. The remaining studies investigating phytosomes in foods focused on the stability of the food, rather than the phytosome integrity (Abdulqahar et al. 2022; Molaveisi et al. 2021; Rabbani et al. 2021).

### 4.2 Stability During Simulated In Vitro Digestion

Human gastrointestinal conditions can be simulated in vitro, with static models often preferred over in vivo approaches due to their reproducibility, ease of standardization, and lower cost. The harmonized INFOGEST 2.0 static digestion model is widely regarded as the standard in vitro digestion method and is commonly used to assess compound bioaccessibility (Brodkorb et al. 2019), defined as the fraction available for absorption in the small intestine after digestion (Verhoeckx et al. 2015). The current study investigated the effects of gastric and intestinal digestion phases with and without enzymes on phenolic stability.

In CSE solutions, the phenolic compounds were generally stable during the gastric phase, both with and without enzymes, consistent with previous reports that phenolics are relatively stable under gastric conditions (Rasera et al. 2023). Most compounds also remained stable during the intestinal phase in the absence of enzymes. The small degree of chemical degradation observed in the intestinal phase could be attributed to the alkaline pH promoting hydroxyl deprotonation and oxidative degradation (Friedman and Jürgens 2000). Their apparent stability was likely due to the short exposure time (2 h) and moderate temperature (37°C).

With respect to the intestinal digestion of CSE in the presence of enzymes, some compounds that were stable at this pH in the absence of enzymes, namely, mangiferin, isomangiferin, and scolymoside, exhibited degradation. In these cases, the digestive enzymes are hypothesized to play a role in their observed stability, as chemical degradation in the absence of enzymes was minimal. Furthermore, the phenolic compounds present in CSE vary in terms of molecular size, solubility, degree of polymerization, and glycosylation, which would influence how they are affected by enzymes (Rasera et al. 2023). It is unclear why the phenolic compounds are more unstable when digestive enzymes are present; C‐ring fission of flavonoids by digestive enzymes has been suggested (Celep et al. 2015). However, based on the literature, phenolic compounds would preferentially bind to the enzymes, which could prevent their degradation. This binding forms insoluble complexes (Martinez‐Gonzalez et al. 2017) and, as a result, phenolic content may be underestimated because these complexes would co‐sediment with the enzymes during centrifugation and are therefore not quantified by liquid chromatography. Subsequent research on the quantification of phenolic compounds at different time points in the intestinal digestion phase, as well as the presence and formation rate of phenolic degradation products, would be required to clarify the mechanisms involved and their kinetics.

Comparison of CSE and CSE‐P digestion showed that most phenolic compounds had similar bioaccessibility in both systems, with slightly lower values for CSE‐P. Conversely, studies that also employed the harmonized INFOGEST 2.0 static digestion protocol reported significantly higher bioaccessibility for phenolic compounds when in a phytosomal formulation (Deleanu et al. 2023; Sahin et al. 2022). The nature of the lipid components, the presence of stabilizers, and the physical properties of the phytosomes may influence their stability during digestion (Campos et al. 2022), suggesting scope for reformulation of CSE‐P. The lipid component and lack of stabilizer likely influenced these outcomes.

### 4.3 Simulated Passive Gastrointestinal Membrane Diffusion of Phenolic Compounds

Artificial cellulose membranes are widely used as alternatives to cell‐free intestinal absorption models for simulating passive diffusion through the small intestinal barrier, which is the primary transport mechanism for phenolics across the gastrointestinal epithelium (Berben et al. 2018; Li et al. 2023).

These experiments are commonly applied to evaluate bioactive release from nanovesicles on the basis that the cellulose membrane allows free bioactive compounds, but not nanovesicles, to diffuse across. This assumes that diffusion across the membrane is instantaneous/nonlimiting and that release from the nanovesicle is the sole rate‐limiting step (Gómez‐Lázaro et al. 2024). However, previous studies have shown that diffusion across the membrane itself significantly delays compound appearance in the receiver compartment (Xie et al. 2015; Yu et al. 2019). Consequently, conventional nanovesicle release models, such as the Gompertz model and others, are unsuitable for describing the measured kinetics, as the observed rate reflects both release from the nanovesicles and diffusion across the membrane. An empirical second‐order kinetic model was therefore applied to determine phenolic passive diffusion rates, where higher rate constants indicate faster diffusion.

The prolonged diffusion of free phenolics in CSE over 7 h confirmed that membrane transport was rate‐limiting. Phenolic compounds are expected to cross the gastrointestinal epithelium primarily by passive diffusion driven by concentration gradients (Li et al. 2023). Faster diffusion during the initial 2–3 h likely reflected the larger initial concentration gradient, with rates decreasing as the gradient diminished over time (Yu et al. 2019). Differences in passive diffusion rates between digestion phases may be explained by pH‐dependent changes in phenolic solubility (Zhang et al. 2023). Variability among compound diffusion may further relate to differences in concentration, molecular size, solubility, diffusivity, partition coefficient, and interactions with other phenolics, consistent with established predictors of gastrointestinal behavior (Lipinski et al. 2001; Veber et al. 2002).

Phenolics in CSE‐P displayed similar overall passive diffusion profiles to free phenolics in CSE, but diffusion was slower. This reduced diffusion rate may first be attributed to lower free phenolic concentrations in the donor phase, as many compounds remain associated with the phytosomal lipid matrix (Wanjiru et al. 2022; Xie et al. 2015). Second, the gradual dissociation of phenolics from the phyto–phospholipid complex likely resulted in prolonged and potentially incomplete release over the 7‐h period (Telange et al. 2021). Such an implied sustained release behavior, which should be confirmed in future studies, may prolong bioactive availability and activity at the target site (Grgić et al. 2020).

## 5 Conclusions

This study demonstrates that phytosomal encapsulation using phospholipid‐based carriers is a viable strategy to improve CSE phenolic compound stability in functional beverage systems. CSE phenolics were more stable in acidified solutions, with pH identified as the most critical determinant of shelf life, particularly for retaining major, high‐value compounds such as mangiferin and PDG. Phytosomal encapsulation provided additional protection during storage, although phytosome quality parameters deteriorated drastically, indicating destabilization of the delivery system despite preservation of bioactive compounds. During simulated gastrointestinal digestion, the expected protective effect of phytosomes was limited, as phenolic stability was comparable between free and encapsulated extracts, suggesting that interactions between phospholipids and digestive enzymes may reduce formulation efficacy and warrant further investigation. Nevertheless, phytosomal encapsulation significantly reduced passive gastrointestinal diffusion rates of phenolics, indicating a prolonged‐release behavior that may prevent rapid exposure in the small intestine and potentially prolong bioactive effects at the target site. In conclusion, while phytosomes effectively enhance the storage stability of CSE phenolics in acidified beverage matrices and modulate their gastrointestinal release, their structural instability during storage and altered behavior during digestion highlight the need for further optimization, particularly focusing on phospholipid–enzyme interactions.

## Author Contributions

**Jade Tobin**: writing – original draft, investigation, data curation. **Chantelle Human**: conceptualization, methodology, writing – review and editing, supervision, funding acquisition. **Dalene de Beer**: writing – review and editing, supervision, methodology. **Marieta van der Rijst**: formal analysis, writing – review and editing.

## Conflicts of Interest

The authors declare no conflicts of interest.

## Supporting information




**Supplementary Material**: jfds71630‐sup‐0001‐TableS1‐S5.pdf
