# Supplementary material for: Phospholipid‐Based Carriers Improve the Stability of Cyclopia subternata Phenolic Compounds in a Beverage Matrix, While Modulating Their Passive Diffusion Across a Simulated Intestinal Membrane
Source: J Food Sci. 2026 Nov 11;91(11):e71630. doi: 10.1111/1750-3841.71630 (PMC13754625; doi:10.1111/1750-3841.71630)
Supplement: Supplementary file 1 [file JFDS-91-0-s001.pdf]

SUPPLEMENTARY MATERIAL

Phospholipid-based carriers improve the stability of *Cyclopia subternata* phenolic compounds in a beverage matrix, while modulating their passive diffusion across a simulated intestinal membrane.

Jade Tobin [a,b], Chantelle Human [a,*], Dalene de Beer [a,b], Marieta van der Rijst [c]

[a] Plant Bioactives Group, Post-Harvest and Agro-Processing Technologies, Agricultural Research Council Infruitec-Nietvoorbij, Private Bag X5026, Stellenbosch, 7599, South Africa
[b] Department of Food Science, Stellenbosch University, Private Bag X1, Matieland (Stellenbosch), 7600, South Africa.
[c] Biometry Unit, Agricultural Research Council, Private Bag X5026, Stellenbosch 7599, South Africa.

TABLE S.1 Simulated gastric fluid (SGF) and simulated intestinal fluid (SIF) at 1.25 × concentration[a].

		SGF (pH 3)[b]		SIF (pH 7)[c]	
	[Stock] mol/L	Volume of stock (mL)	[SGF] (mmol/L)	Volume of stock (mL)	[SIF] (mmol/L)
KCl	0.50	17.25	6.90	17.00	6.80
KH_2PO_4	0.50	2.25	0.90	2.00	0.80
NaCl	2.00	45.13	72.20	77.00	123.20
$MgCl_2(H_2O)_6$	0.15	1.00	0.12	2.75	0.33
$(NH_4)_2CO_3$	0.50	1.25	0.50	n/a	n/a
HCl	6.00	3.25	15.60	1.75	8.40

[a]All constituents were combined and the mixtures made up to 1000 mL using deionised water; [b-c]The pH of the SGF and SIF was adjusted to 3 using 0.1 M HCl and 7 using 0.1 M NaOH, respectively. *Abbreviations*: KCl, potassium chloride; KH_2PO_4, potassium dihydrogenphosphate; NaCl, sodium chloride; $MgCl_2(H_2O)_6$, magnesium hexahydrate; $(NH_4)_2CO_3$, ammonium carbonate; HCl, hydrochloric acid; n/a, not applicable.

TABLE S.2 Example of a simulated *in vitro* gastrointestinal digestion experiment for the *Cyclopia subternata* extract (CSE) in simulated gastric and intestinal digestion fluids with and without enzymes. Ratios of constituents are based on Brodkorb *et al.* (2019).

	**With enzymes/bile extract**			**Without enzymes/bile extract**		
**Description**	**Gastric phase**	**Intestinal phase**		**Gastric phase**	**Intestinal phase**	
CSE solution/gastric digesta (mL)	2	4.025		2	4.025	
Electrolyte solutions (mL)	1.6	1.6		1.6	3.5	
Enzymes	pepsin	trypsin in pancreatin	bile acid	pepsin	trypsin in pancreatin	bile acid
Required enzyme activity (U/mL) or bile acid concentration (mM) in total digesta[a]	2000	100	10	n/a	n/a	n/a
Specific activity (U/mg) or bile acid concentration (mmol/g)[b]	11437	161	2.793	n/a	n/a	n/a
Concentration of enzyme/bile extract solution (mg/mL)[c]	10.05	10.00	20.08	n/a	n/a	n/a
Volume of enzyme/bile to be added (mL)[d]	0.070	0.497	1.427	n/a	n/a	n/a
Water (mL)[e]	0.330	0.451		0.400	0.451	
Target pH	3	7		3	7	
HCl (0.1 M) for pH adjustment (mL)[f]	0.025	n/a		0.025	n/a	
NaOH (0.1 M) for pH adjustment (mL)[f]	n/a	0.066		n/a	0.066	
Final volume (mL)	4.025	8.066		4.025	8.066	
Dilution factor	2.013	4.033		2.013	4.033	

[a]Required enzyme activity and bile acid concentration values sourced from Brodkorb *et al.* (2019) based on enzyme activity in the human digestive tract; [b]specific enzyme activity determined using enzyme assays; [c]concentration of enzymes/bile extract based on exact mass weighed per replicate experiment; [d]volume of enzyme/bile extract solution calculated based on the specific enzyme activity/bile acid concentration and enzyme/bile extract solution concentration, adjusted for the total final volume; [e]the volume of water added is adjusted to obtain a volume of 4 or 8 mL before pH adjustment; [f]the volume of HCl or NaOH added is adjusted based on the specific sample. *Abbreviation*: n/a, not applicable.

TABLE S.3. The adjusted coefficients of determination (R^2_{adj}) between measured and predicted content for the first-order model[a] for quantified phenolic compound degradation during storage at 40°C for 90 days, respectively. Results are reported for *Cyclopia subternata* extract (CSE) and CSE-loaded phytosomes (CSE-P).

Model solution	IDG		Eriocitrin		PDG		Hesperidin		Mangiferin		Isomangiferin		Scolymoside	
	CSE	CSE-P	CSE	CSE-P	CSE	CSE-P	CSE	CSE-P	CSE	CSE-P	CSE	CSE-P	CSE	CSE-P
**pH 3**	0.9794	0.8901	0.9432	0.9248	0.9904	0.9008	0.9867	0.7514	0.9558	0.9683	0.9915	0.9665	0.9615	0.8439
**pH 5**	0.7866	0.8034	0.9822	0.9904	0.976	0.9957	0.9487	0.8885	0.9967	0.9959	0.9854	0.9953	0.9713	0.9833
**pH 7**	0.6531	0.8083	nd	nd	nd	nd	0.9987	0.9768	nd	nd	nd	nd	0.9954	0.9777

[a]First-order model: $C = C_0(\exp(-kt))$ where *C* is the concentration of the compound (g/100 g extract), C_0 the initial concentration of the compound (g/100 g extract), t the time (days) and *k* is the reaction rate constant. *Abbreviations*: IDG, 3-β-D-glucopyranosyl-4-*O*-β-D-glucopyranosyliriflophenone; PDG, 3′,5′-di-β-D-glucopyranosylphloretin; nd, not determined.

TABLE S.4 Passive diffusion (%, mean ± SD) of phenolic compounds over time into the receiver compartment containing simulated gastric and intestinal fluids (without enzymes) during simulated passive gastrointestinal membrane diffusion using *Cyclopia subternata* extract (CSE) and CSE-loaded phytosomes (CSE-P).

Model solution	Digestion phase	Time (h)	IDG	Eriocitrin	PDG	Hesperidin	Mangiferin	Isomangiferin	Scolymoside
CSE	Gastric	0	0.0v ± 0.0	0.0q ± 0	0.0t ± 0.0	0.0s ± 0.0	0.0w ± 0.0	0.0t ± 0.0	0.0s ± 0.0
		0.25	11.2s ± 2.7	10.1p ± 2.9	8.8q ± 2.7	7.7p ± 2.6	13.7s ± 2.9	1.7st ± 1.5	6.8r ± 2.5
		0.5	19.4p ± 2.6	18.4m ± 2.8	16.5o ± 2.4	15.5n ± 2.3	24.9p ± 3.6	11.2p ± 3.5	14.9no ±3.9
		1	43.3j ± 2.4	43.4i ± 3.5	40.9i ± 2.7	39.0i ± 1.8	51.3j ± 2.8	39.1j ± 3.6	35.5i ± 3.4
		2	50.2h ± 2.1	50.1gh ± 2.1	48.3h ± 2.4	46.5g ± 2.1	60.6g ± 2.3	47.8h ± 3.6	43.3g ± 2.9
		3	60.9e ± 1.0	60.1fg ± 0.3	60.2e ± 1.3	57.5e ± 1.2	69.7e ± 1.1	56.5e ± 2.9	51.9d ± 2.4
		5	74.8b ± 0.5	75.0c ± 0.6	74.6c ± 2.5	73.1c ± 2.0	82.7a ± 1.4	69.4b ± 2.8	66.4b ± 3.1
		7	81.7a ± 0.9	81.3a ± 1.9	82.5a ± 0.8	80.4a ± 2.0	85.9a ± 1.4	73.6a ± 3.1	71.0a ± 2.0
	Intestinal	0	0.0v ± 0.0	0.0q ± 0.0	0.0t ± 0.0	0.0s ± 0.0	0.0w ± 0.0	0.0t ± 0.0	0.0s ± 0.0
		0.25	9.1t ± 2.2	9.3p ± 2.0	8.3q ± 2.2	7.1pq ± 2.2	12.4st ± 2.1	0.2t ± 0.5	5.5r ± 1.8
		0.5	16.8q ± 2.8	17.7m ± 2.6	16.3o ± 2.2	14.4n ± 1.9	22.3q ± 2.1	8.2q ± 2.2	11.8pq ± 1.8
		1	39.4k ± 2.6	42.0i ± 1.3	39.8ij ± 1.3	37.4ij ± 0.7	49.8kj ± 1.0	35.9k ± 1.2	32.0j ± 1.0
		2	46.6i ± 2.1	48.4h ± 2.1	48.0h ± 1.0	45.6g ± 1.2	56.6h ± 1.8	43.4i ± 1.9	37.5i ± 3.3
		3	57.3f ± 2.0	61.2de ± 1.4	59.9ef ± 1.0	57.1e ± 0.7	67.7f ± 1.2	53.9f ± 1.5	47.3e ± 1.9
		5	67.8d ± 2.4	72.9c ± 0.6	71.8d ± 0.9	69.9d ± 1.0	76.9d ± 1.0	62.7d ± 1.3	58.9c ± 2.7
		7	70.7c ± 1.8	77.9b ± 1.6	77.8b ± 2.1	75.4b ± 2.3	80.8c ± 1.7	66.4c ± 2.3	64.3b ± 2.0
CSE-P	Gastric	0	0.0v ± 0.0	0.0q ± 0.0	0.0t ± 0.0	0.0s ± 0.0	0.0w ± 0.0	0.0t ± 0.0	0.0s ± 0.0
		0.25	8.7t ± 1.6	13.2no ± 1.4	6.3r ± 1.7	5.6q ± 1.4	9.4u ± 1.3	2.5s ± 1.1	11.3pq ± 0.9
		0.5	14.9r ± 2.0	18.5m ± 1.6	11.7p ± 2.0	11.0o ± 1.6	16.0r ± 1.4	9.2q ± 1.3	15.9n ± 0.5
		1	28.1n ± 1.2	31.0k ± 0.9	24.3m ± 0.6	23.0l ± 1.0	30.6n ± 1.0	24.3m ± 0.3	26.3l ± 0.3
		2	33.8l ± 3.4	36.4j ± 2.6	30.4l ± 3.3	28.8k ± 3.2	36.6m ± 2.9	30.2l ± 2.1	30.9jk ± 2.1
		3	40.9k ± 3.7	43.6i ± 2.9	38.0j ± 4.6	35.6j ± 3.3	43.6l ± 3.0	37.4jk ± 2.5	36.8i ± 2.2
		5	49.9h ± 4.7	54.2f ± 3.7	48.7h ± 4.3	46.1g ± 3.6	54.1i ± 2.2	48.3h ± 1.4	45.9ef ± 1.2
		7	53.5g ± 0.4	59.1e ± 0.8	53.9g ± 0.7	51.5f ± 0.7	59.3g ± 0.8	54.4f ± 0.3	51.5d ± 0.8
	Intestinal	0	0.0v ± 0.0	0.0q ± 0.0	0.0t ± 0.0	0.0s ± 0.0	0.0w ± 0.0	0.0t ± 0.0	0.0s ± 0.0
		0.25	4.6u ± 0.4	11.0op± 0.9	3.9s ± 0.4	3.1r ± 0.5	6.5v ± 0.8	0.1t ± 0.2	10.5q ± 0.2
		0.5	8.2t ± 1.1	14.9n ± 1.3	8.4q ± 0.4	6.7pq ± 0.8	11.3t ± 0.7	4.4r ± 0.6	12.9op ± 1.6
		1	18.5pq ± 1.9	26.7l ± 1.3	20.8n ± 0.9	18.2m ± 0.8	24.2p ± 0.6	17.6o ± 0.6	22.0m ± 0.4
		2	23.7o ± 0.8	32.4k ± 1.5	25.6m ± 1.5	22.6l ± 1.3	27.9o ± 0.9	21.6n ± 1.0	25.5l ± 1.8
		3	31.4m ± 1.2	38.7j ± 2.7	33.8k ± 1.9	28.7k ± 1.9	36.0m ± 1.1	29.4l ± 0.6	29.7k ± 1.5
		5	44.9ij ± 1.7	51.7g ± 2.2	47.2h ± 3.0	43.3h ± 1.5	49.0k ± 1.5	42.0i ± 0.8	40.0h ± 2.2
		7	53.1g ± 1.8	62.1d ± 1.9	58.0f ± 3.6	53.0f ± 2.1	56.0h ± 2.1	50.1g ± 2.4	44.7fg ± 1.3

[a]Sum of quantified phenolic content. *Abbreviations*: PDG, 3′,5′-di-β-D-glucopyranosylphloretin; IDG, 3-β-D-glucopyranosyl-4-*O*-β-D-glucopyranosyliriflophenone; HPDG, 3′,5′-di-β-D-glucopyranosyl-3-hydroxyphloretin. Values for a compound with different letters are significantly different ($p < 0.05$).

TABLE S.5 The adjusted coefficients of determination (R^2_{adj}) between measured and predicted passive diffusion for phenolic compounds into the receiver compartment containing simulated gastric and intestinal fluids (without enzymes) during simulated passive gastrointestinal membrane diffusion, for the second-order model. Results are reported for *Cyclopia subternata* extract (CSE) and CSE-loaded phytosomes (CSE-P).

Compound	Samples	Digestion phase	Second-order[a]
IDG	CSE	Gastric	0.9865
		Intestinal	0.9811
	CSE-P	Gastric	0.9634
		Intestinal	0.9825
Eriocitrin	CSE	Gastric	0.9813
		Intestinal	0.9781
	CSE-P	Gastric	0.9655
		Intestinal	0.9771
PDG	CSE	Gastric	0.9856
		Intestinal	0.9882
	CSE-P	Gastric	0.9797
		Intestinal	0.9872
Hesperidin	CSE	Gastric	0.9851
		Intestinal	0.9851
	CSE-P	Gastric	0.9776
		Intestinal	0.9844
Mangiferin	CSE	Gastric	0.9873
		Intestinal	0.9748
	CSE-P	Gastric	0.9704
		Intestinal	0.9812
Isomangiferin	CSE	Gastric	0.9678
		Intestinal	0.9481
	CSE-P	Gastric	0.9726
		Intestinal	0.9825
Scolymoside	CSE	Gastric	0.9832
		Intestinal	0.9547
	CSE-P	Gastric	0.9510
		Intestinal	0.9456

[a]Second-order model: $C = C_0/(1 + C_0kt)$ where *C* is the diffusion of the compound into the receiver compartment at time t (hours), C_0 is the initial concentration (100 %) at t = 0 h and *k* is the passive diffusion rate constant; [c]sum of quantified phenolic content. *Abbreviations*: IDG, 3-β-D-glucopyranosyl-4-*O*-β-D-glucopyranosyliriflophenone; 3′,5′-di-β-D-glucopyranosyl-3-hydroxyphloretin; PDG, 3′,5′-di-β-D-glucopyranosylphloretin.
